# Prognostic Stratification in Primary Glomerulonephritis: Integrating Histology, Biomarkers, and Risk Prediction Models

**DOI:** 10.3390/life16030419

**Published:** 2026-03-04

**Authors:** Andreea Simona Covic, Adrian Covic, Irina Draga Caruntu, Lucian Siriteanu, Mehmet Kanbay, Gener Ismail, Luminița Voroneanu, Mihai Onofriescu

**Affiliations:** 1 Department of Nephrology, “Grigore T. Popa” University of Medicine and Pharmacy, 700115 Iași, Romania; andreea.covic@gmail.com (A.S.C.); siriteanulucian@gmail.com (L.S.); lumivoro@yahoo.com (L.V.); onomihai@yahoo.com (M.O.); 2 “Dr. C. I. Parhon” University Hospital, 700503 Iași, Romania; irinadragacaruntu@gmail.com; 3 Romanian Medical Science Academy, 030171 Bucharest, Romania; 4 Department of Pathology, “Grigore T. Popa” University of Medicine and Pharmacy, 700115 Iași, Romania; 5 Department of Medicine, Division of Nephrology, Koc University School of Medicine Istanbul, Sarıyer 34450, Turkey; mkanbay@ku.edu.tr; 6 Department of Nephrology, “Carol Davila” University of Medicine and Pharmacy, 050474 Bucharest, Romania; gener732000@yahoo.com; 7 Fundeni Clinical Institute, 022328 Bucharest, Romania

**Keywords:** primary glomerulonephritis, prognostic biomarkers, histologic scoring systems, risk prediction models, renal outcome

## Abstract

Primary glomerulonephritis encompasses a diverse group of kidney diseases with variable clinical trajectories and outcomes. Accurate prognostic stratification is critical for guiding individualized management and improving long-term renal survival. This narrative review synthesizes current evidence on the prognostic value of histological grading systems, circulating and urinary biomarkers, and integrative risk prediction models across major primary glomerulonephritis subtypes, including IgA nephropathy, membranous nephropathy, and focal segmental glomerulosclerosis. Emphasis is placed on the utility of established classification systems (e.g., Oxford, MEST-C, chronicity scores), emerging tissue and fluid biomarkers (e.g., PLA2R antibodies, complement components, cytokine profiles), and the validation of multivariable prognostic tools and nomograms. We highlight areas of convergence between histopathologic lesions and molecular markers, as well as the evolving role of machine learning in predictive modeling. Ultimately, combining morphological, biochemical, and algorithmic tools holds promise for precision risk assessment and treatment tailoring in primary glomerulonephritis.

## 1. Introduction

Primary glomerulonephritis (GN) encompasses a heterogeneous group of immune-mediated kidney diseases that remain a leading cause of chronic kidney disease and end-stage kidney failure worldwide [1]. Despite advances in disease classification and the increasing availability of targeted therapies, predicting long-term renal outcomes at the individual patient level remains challenging. Traditional prognostic assessment has relied largely on baseline clinical parameters—such as proteinuria and estimated glomerular filtration rate—yet these variables incompletely capture the biological diversity and dynamic course of glomerular diseases. Over the past two decades, a growing body of evidence has demonstrated that histologic features on kidney biopsy, particularly markers of chronicity and irreversible structural damage, provide powerful prognostic information that extends beyond diagnosis alone. In parallel, disease-specific serological and urinary biomarkers, genetic risk factors, and longitudinal clinical measures have emerged as important modifiers of outcome and treatment response. More recently, these diverse prognostic domains have been integrated into composite risk scores, nomograms, and machine-learning-based models, aiming to refine individualized risk stratification and support precision-based therapeutic decision-making. This narrative review synthesizes current evidence on histological, clinical, biochemical, and computational prognostic tools across major forms of primary glomerulonephritis, highlighting shared principles, disease-specific nuances, and remaining gaps in prognostic assessment.

## 2. Materials and Methods

A targeted literature search was performed in PubMed/MEDLINE and Embase to identify studies published over the last 10 years (January 2015–September 2025) addressing prognostic stratification in primary glomerulonephritis, with emphasis on membranous nephropathy, IgA nephropathy, focal segmental glomerulosclerosis/minimal change disease, and C3 glomerulopathy/MPGN. Search terms combined disease-specific keywords with prognostic concepts, including “prognosis”, “renal outcome”, “risk prediction”, “histologic score”, “biomarker”, “PLA2R”, “complement”, “nomogram”, and “machine learning”. Reference lists of key meta-analyses, multicenter cohorts, and guideline documents were also screened.

Priority was given to systematic reviews/meta-analyses, large registry studies, multicenter cohorts, randomized trials and post hoc analyses, and externally validated prognostic models. Studies were excluded if they primarily involved secondary glomerular diseases, pediatric-only populations, animal/preclinical research, case reports, congress abstracts, or non-English publications. Article selection and synthesis were performed by the authors based on relevance, methodological robustness, and clinical applicability, with consensus discussion for inclusion of key evidence.

## 3. Membranous Nephropathy

### 3.1. Histological Prognostic Factors

#### 3.1.1. Glomerular Lesions: Focal Segmental Glomerulosclerosis and Segmental Lesions

Focal segmental glomerulosclerosis (FSGS) is a consistent histological predictor of adverse renal outcomes in membranous nephropathy (MN). In a large national registry (n = 752), FSGS was independently associated with lower eGFR, higher proteinuria, and reduced remission rates, and was incorporated into the composite FSTIV score together with tubular atrophy (TA), interstitial fibrosis (IF), and vascular hyalinosis [2]. Correspondingly, in a cohort of 716 patients, typical FSGS lesions independently predicted ≥50% eGFR decline or end stage kidney disease (ESKD) (HR 2.47), while atypical segmental lesions were not prognostic [3]. Event-free survival was significantly lower in patients with typical FSGS lesions.

#### 3.1.2. Tubulointerstitial Injury

Tubulointerstitial damage (TID) consistently correlates with worse renal outcomes in MN. Across several cohorts (n = 300–582), the presence and severity of TID were associated with higher baseline proteinuria and creatinine, lower remission rates, and poorer renal survival [4,5]. Severe chronic TID (>50%) emerged as a strong independent predictor of ESKD (HR 25.77) and significant eGFR decline [6]. Although not uniformly predictive of remission in multivariable analyses, the burden of tubulointerstitial injury appears to reflect irreversible structural damage and long-term risk.

#### 3.1.3. Vascular Lesions: Arteriolosclerosis

Arteriolar changes are emerging prognostic markers in MN. In a 10-year cohort (n = 597), patients with arteriolar lesions had a significantly higher rate of composite renal events (23.9% vs. 11.2%) [7]. Independent predictors included age, elevated serum creatinine (SCr), IgM (3+) deposits, and IF. Interestingly, C1q (3+) and TA ≥ 50% were associated with paradoxical protective effects, possibly reflecting adaptive mechanisms [7].

#### 3.1.4. Basement Membrane Alterations

Ultrastructural findings provide additional prognostic insight. Increased glomerular basement membrane (GBM) thickness was independently associated with a reduced likelihood of complete remission (HR per SD, 0.58) and correlated with greater complement and PLA2R deposition [8]. Advanced Ehrenreich–Churg stages were also independently linked to reduced single-nephron GFR (SNGFR), supporting a functional impact of ultrastructural GBM remodelling beyond global kidney function measures [9]. Conventional eGFR does not capture nephron reserve or compensatory hyperfiltration, whereas SNGFR may better reflect functional nephron workload.

### 3.2. Clinical and Biochemical Prognostic Factors

#### 3.2.1. Serological Prognostic Biomarkers: Anti-PLA2R Antibodies

Anti-PLA2R antibodies represent the most robust serological prognostic marker in idiopathic MN. A meta-analysis including 1761 patients demonstrated that seropositivity was associated with lower complete and spontaneous remission rates (OR 0.50 and 0.30, respectively) [10]. Higher antibody titers further reduced the probability of remission, although no consistent association with progression to ESKD was observed. Seronegative patients in Asia showed higher spontaneous remission, and CR was more likely in PLA2R-negative individuals treated with calcineurin inhibitors for 3–6 months [10]. These findings support anti-PLA2R status as a marker of immunologic activity and treatment response rather than as a marker of long-term renal survival per se.

#### 3.2.2. Urinary Biomarkers

*Hematuria*. In a large cohort (n = 639), baseline and persistent microscopic hematuria independently predicted relapse and renal progression. Persistent baseline hematuria was associated with a 52% higher risk of relapse (HR = 1.52, 95% CI: 1.02–2.29), and worsening hematuria during follow-up increased the short-term relapse risk almost fivefold (adjusted HR = 4.64, 95% CI: 3.29–6.54) [11]. Time-averaged hematuria and longer cumulative exposure were associated with ≥40% eGFR decline or ESKD, whereas hematuria remission was associated with improved outcomes [11].

*Low-molecular-weight proteins*. Urinary β2-microglobulin and α1-microglobulin showed good predictive accuracy for renal progression (AUC ≈ 0.80), particularly in patients with preserved baseline kidney function, suggesting their role as markers of tubular injury [12].

*Acute kidney injury*. AKI during follow-up is associated with significantly lower renal survival and a higher incidence of ESKD, even in the absence of baseline histologic differences [13].

*Exploratory biomarkers (cytokine profiles)*. Post hoc analysis of the STARMEN trial identified cytokine profiles associated with treatment response. Lower baseline urinary CXCL13 predicted remission under tacrolimus–rituximab, while decreasing GDF15 levels were linked to a promising response to corticosteroid–cyclophosphamide therapy [14]. Additionally, several cytokines—plasma and urinary GDF15, plasma TNF-α, and urinary TWEAK—correlated significantly with markers of disease activity. Although exploratory, these findings suggest potential immunophenotypic stratification in MN.

Table 1 summarizes evidence from cohort studies and post hoc clinical trial analyses evaluating histologic and biomarker-based predictors of outcome in membranous nephropathy. Endpoints include remission, renal function decline, ESKD, and treatment response, with effect estimates reported where available.

### 3.3. Nomograms and Risk Scores for Prognosis

Several prognostic tools have been developed to predict outcomes in idiopathic MN, using a range of biochemical, clinical, and pathological parameters (Table 2).

#### Clinical Scores

*The Toronto Risk Score*, based on longitudinal proteinuria and creatinine trajectories, remains widely used (AUC ≈ 0.78) [12]. A new score incorporating age, eGFR, and proteinuria (formula: Risk score = 0.04 × Age (years) − 0.03 × eGFR + 0.09 × proteinuria (g/24 h) demonstrated strong discrimination for progression (AUC 0.83) [15]. Each one-point increase was associated with a 2.57-fold higher risk of progression to a composite endpoint (≥30% eGFR decline, ESKD, or death).

*Biochemical-enhanced models*. The fibrinogen-to-albumin ratio (FAR) independently predicted non-remission, with a cutoff of 0.233 identifying patients at increased risk (multivariate HR 4.00). FAR correlated with proteinuria and anti-PLA2R titers and demonstrated moderate discrimination (AUC 0.738), which modestly improved when combined with anti-PLA2R antibodies (AUC 0.766) [16].

*Machine learning models*. A 2023 study applied five machine learning algorithms to electronic medical record data from 418 biopsy-proven idiopathic MN cases to improve individualized risk prediction [17]. The LightGBM model showed the best performance (AUC 0.892), with anti-PLA2R levels, IgG4 staining, β2-microglobulin, D-dimer, triglycerides, albumin, AST, serum creatinine, and fasting glucose identified as key predictors. High anti-PLA2R and low IgG4 were most strongly associated with adverse outcomes, highlighting the potential of ML-based approaches for individualized risk stratification [17].

*Nomograms*. Three recent studies developed nomogram models for individualized prognosis in idiopathic MN. These incorporated variables, such as anti-PLA2R titers, albumin, proteinuria (including time-averaged values), disease duration, D-dimer, and sPLA2R antibodies, achieved AUCs ranging from 0.80 to 0.87 [18,19,20]. The best-performing model, a random forest, demonstrated excellent discrimination (AUC 0.869) in both internal and external validation [20]. Collectively, these tools demonstrate favorable discrimination and potential clinical applicability, although broader validation remains essential.

Table 2 summarizes established and emerging risk prediction models for membranous nephropathy integrating clinical, biochemical, histologic, and serologic variables. Model performance is reported as AUC. The LightGBM model represents the application of machine learning-based approaches to individualized risk prediction.

## 4. Membranoproliferative Glomerulonephritis

### 4.1. Histological Prognostic Factors

Recent multicenter cohort studies indicate that chronic structural lesions, rather than immunopathologic subtype, are the principal determinants of renal outcome in C3 glomerulopathy (C3G) and idiopathic immunoglobulin-associated MPGN [21,22,23,24]. Across international cohorts, interstitial fibrosis/tubular atrophy (IF/TA), segmental glomerulosclerosis, and cellular or fibrocellular crescents consistently predicted composite renal endpoints, including significant eGFR decline, doubling of serum creatinine, and ESKD. Lower baseline eGFR was strongly associated with chronic histologic damage, whereas endocapillary hypercellularity and GBM double contours correlated primarily with proteinuria severity.

The C3G Histologic Index further quantified activity and chronicity, demonstrating that chronic lesions—particularly glomerulosclerosis and IF/TA—are the strongest predictors of progression [23]. The Total Chronicity Score (TCS) incorporates glomerulosclerosis, interstitial fibrosis, tubular atrophy, and vascular sclerosis [22]. A TCS ≥ 4 was associated with lower 3-year renal survival (72% vs. 91%) in a multicenter study of 74 patients with C3G; it did not remain an independent predictor after multivariable adjustment, and lower haemoglobin emerged as the only independent predictor of outcome [22,23].

Importantly, comparative analyses show minimal prognostic difference between C3GN and dense deposit disease (DDD), or between C3G and immune complex-mediated MPGN, indicating that total histologic burden and chronicity provide greater prognostic discrimination than etiologic subclassification alone [23,24]. In a multicenter U.S. cohort of 111 patients, C3GN showed higher activity scores and DDD greater chronicity; however, renal outcomes did not differ significantly between subtypes [23]. Decreased eGFR and chronic lesions—particularly interstitial fibrosis and glomerulosclerosis—were the strongest predictors of progression, and both activity and chronicity indices independently predicted renal deterioration. Similar findings from a Finnish cohort confirmed minimal prognostic differences between immune-complex-mediated MPGN and C3G, underscoring the greater relevance of overall histologic burden than subtype classification [24].

### 4.2. Clinical and Biochemical Prognostic Factors

*Proteinuria* remains the principal dynamic clinical predictor of progression. Longitudinal analyses demonstrate that doubling of proteinuria is associated with approximately 2.5-fold increased risk of renal insufficiency, whereas ≥50% reduction—particularly within the first 6–12 months—confers significantly improved renal outcomes [25,26]. Even modest early reductions (>30% at 6 months) are associated with better renal survival, and sustained proteinuria < 1 g/day correlates with improved long-term prognosis (26). Baseline eGFR independently predicts progression across cohorts, reinforcing the interaction between initial renal function and cumulative structural injury.

*Complement biomarkers*, including low-serum C3, elevated soluble C5b-9, and high C3 nephritic factor activity, identify biologically active complement dysregulation and are associated with more rapid progression [27]. However, their prognostic effect appears largely mediated through acceleration of structural damage rather than independent prediction of outcome.

Collectively, chronic histologic injury and dynamic clinical indices—particularly proteinuria trajectory and baseline eGFR—form the core framework for risk stratification in C3G/MPGN.

Table 3 summarizes histologic and clinical predictors of renal outcome in C3G derived from cohort studies and multicenter registries. Chronic lesions—particularly interstitial fibrosis, tubular atrophy, and segmental sclerosis—consistently predict progression, whereas longitudinal reduction in proteinuria is associated with improved prognosis. Quantitative histologic indices (e.g., chronicity and activity scores) and baseline eGFR further refine risk stratification.

Table 4 synthesizes comparative data across histopathologic subtypes of C3G and immune complex-mediated MPGN, demonstrating limited prognostic discrimination between entities such as C3GN and DDD; the overall structural burden appears more informative than etiologic subclassification.

Table 5 outlines complement-related biomarkers associated with adverse outcomes in C3G. Low-serum C3, elevated soluble C5b-9, and increased C3 nephritic factor activity correlate with more rapid progression and may provide adjunctive value in risk assessment and disease monitoring.

## 5. IgA Nephropathy

### 5.1. Histological Prognostic Factors

The Oxford Classification (MEST-C) remains the cornerstone of histologic risk stratification in IgA nephropathy (IgAN) [28]. A large meta-analysis of 16 retrospective cohorts, including 3893 patients with biopsy-proven IgAN (570 kidney failure events), evaluated the prognostic performance of the Oxford lesions [29]. Kidney failure was defined as doubling of serum creatinine, ≥50% eGFR decline, or ESKD. In multivariable models, M, S, and particularly T lesions independently predicted adverse outcomes (HR 1.8 for S1; HR 3.2 for T1/T2), whereas E was not independently associated with progression and showed substantial heterogeneity [29]. Crescentic lesions, initially excluded from the original classification, were subsequently incorporated into the MEST-C score after consistent evidence demonstrated their association with adverse renal outcomes [30,31,32]. Meta-analyses consistently demonstrate that crescent formation is associated with increased risk of kidney failure in IgAN (HR ≈1.9–2.0) [30,31]. Risk rises in a dose–response manner with crescent burden, particularly when ≥25% of glomeruli are involved, independent of immunosuppressive therapy, supporting crescents as markers of severe disease and adverse prognosis [31,32].

The prognostic relevance of the Oxford (MEST-C) classification has also been confirmed in pediatric IgAN. In a meta-analysis of 14 cohort studies including 5679 children, M1, S1, and T1/T2 were independently associated with progression, with S and T lesions showing the strongest effects [33]. Crescentic lesions (C1/C2) were likewise associated with adverse outcomes across ethnic groups, whereas E1 demonstrated inconsistent associations. These findings align with adult data, supporting chronic lesions—particularly S and T—as primary determinants of long-term prognosis, whereas active lesions appear to be treatment-dependent. Interobserver reproducibility of the Oxford lesions is variable, with the highest for T and the lowest for E and C [34]. T shows the most consistent independent prognostic value, whereas associations for E and C are heterogeneous, particularly in immunosuppressed cohorts.

To improve global risk stratification, the individual MEST-C components were summed into a cumulative Oxford score (range 0–7), allowing classification into three O-grades [35]. In 871 patients, higher O-grades were associated with progressively lower 10-year renal survival (94.1%, 86.9%, and 74.1% for grades I–III), with O-grade II and III conferring increased risk of adverse outcomes (HR 2.8 and 6.3), independent of clinical adjustment; prognostic discrimination further improved when combined with clinical classification. Because IgAN biopsies may contain a limited number of glomeruli, histologic classification can be affected by sampling variability and may not reliably reflect true lesion burden. To address this limitation, a Bayesian probabilistic approach was applied to quantify diagnostic confidence in the Japanese histologic classification when glomerular yield is low. The analysis showed that even in biopsies with ≤9 glomeruli, high posterior probabilities (≥90%) for correct classification could still be achieved, suggesting that probabilistic modeling may help support the reliability of prognostic grading in suboptimal biopsy specimens [36].

Additional refinements include global injury pattern classification and mesangial complement deposition. Sclerosing and crescentic patterns independently predict faster eGFR decline (2.1-fold and 3.6-fold increased risk of adverse outcomes compared with the mesangioproliferative pattern), and intense mesangial C3 deposition confers increased progression risk (HR ≈ 3.3), suggesting additive prognostic value beyond conventional scoring [37].

### 5.2. Clinical and Biochemical Prognostic Factors

Sustained proteinuria remains the dominant clinical predictor of progression in IgAN. Large registry data from 2439 patients in the UK National Registry of Rare Kidney Diseases (RaDaR) cohort demonstrated a strong association between time-averaged proteinuria and kidney failure risk, with substantial progression observed even at low proteinuria levels (<1 g/day)—with approximately 30% of patients with time-averaged proteinuria 0.44–0.88 g/g and ~20% with <0.44 g/g developing kidney failure within 10 years [38]. Each 10% reduction in time-averaged proteinuria was associated with a 11% lower risk of kidney failure or death (HR 0.89), and the lifetime risk remained high unless eGFR decline was maintained at ≤1 mL/min/1.73 m^2^/year.

Similarly, time-weighted average proteinuria (TWAP) independently predicted accelerated eGFR decline in 549 patients. In those with baseline eGFR 15–60 mL/min/1.73 m^2^, higher TWAP demonstrated a graded association with faster annual eGFR loss (approximately −2.0, −3.4, and −4.0 mL/min/1.73 m^2^/year across increasing strata), whereas in patients with preserved kidney function (eGFR ≥ 60 mL/min/1.73 m^2^), significant acceleration was observed only at TWAP ≥1 g/g (≈−5.7 mL/min/1.73 m^2^/year) [39].

Together, these data demonstrate a dose–response relationship between sustained proteinuria and eGFR decline, supporting cumulative proteinuria metrics as key prognostic markers and reinforcing maintenance of proteinuria < 0.3 g/g.

Early changes in kidney function trajectory have emerged as key prognostic markers in IgAN. In a meta-analysis of 1299 patients from 13 randomized trials, treatment effects on 1-year eGFR slope strongly correlated with long-term renal outcomes (R^2^ = 0.86), whereas 1-year proteinuria reduction showed weaker and non-independent associations [40]. One-year eGFR slope remained an independent predictor after adjustment, supporting its role as a validated surrogate endpoint for risk stratification and clinical trials.

In a meta-analysis of 13 cohort studies including 5660 patients, hematuria at diagnosis was not independently associated with ESKD overall [41]. Initial microscopic hematuria was associated with increased risk (RR 1.87), whereas macroscopic hematuria was inversely associated with ESKD; persistent hematuria during follow-up emerged as a potential independent predictor of adverse renal outcomes, including ESKD or ≥50% eGFR decline. Table 6 summarizes evidence from meta-analyses and large cohort studies on histologic and clinical predictors of progression in IgAN. MEST-C components, particularly M1, S1, and T1/T2, remain central to risk stratification, with additional prognostic value for crescent burden and global injury patterns. Among clinical markers, cumulative proteinuria, eGFR slope, and persistent hematuria are key determinants of progression, with 1-year eGFR slope serving as a validated surrogate endpoint in clinical trials.

### 5.3. Nomograms and Risk Scores for Prognosis

The International IgAN Prediction Tool represents the most extensively validated disease-specific model for risk stratification in IgAN [42]. Derived from 3927 patients and externally validated in another 1146 patients, it integrates clinical variables (eGFR, blood pressure, proteinuria) and MEST-C lesions to predict ≥50% eGFR decline or ESKD, achieving C-statistics of approximately 0.81–0.82 and demonstrating good calibration across multi-ethnic cohorts. Versions with and without race performed similarly [42].

External validations in independent cohorts (n ≈ 2300) confirmed comparable discrimination (C ≈ 0.81–0.82) for composite renal outcomes, with performance exceeding 0.90 for ESKD prediction [43]. Models incorporating histologic variables showed improved discrimination in patients with preserved kidney function. In older adults—similar discrimination for both race-based and race-free versions (C ≈ 0.79) over a median 5.1-year follow-up [44]. Calibration differed modestly, with the race-free model showing slightly improved 5-year risk estimation, although reclassification gains were minimal.

A systematic review comparing the Kidney Failure Risk Equation (KFRE) with Oxford-based IgAN-specific models identified a single eligible cohort (n = 2300) and showed similar discrimination for ESKD prediction (C ≈ 0.90–0.91), with slightly better performance for the IgAN-specific model in patients with preserved kidney function; overall certainty was limited by risk of bias and short follow-up [45].

External validation of the 4-variable KFRE in 236 patients with IgAN and CKD stage 3 demonstrated fair short-term discrimination (AUC 0.78 at 5 years) but weaker long-term performance (AUC 0.64 at 20 years); recalibration improved discrimination and model fit, supporting disease-specific adaptation for long-term risk prediction in IgAN [46].

Table 7 summarizes prognostic models for kidney failure risk in IgAN. The International IgAN Prediction Tool is the most extensively validated, integrating clinical and histologic variables with consistent performance across diverse cohorts. Generic models such as the KFRE demonstrate acceptable short-term discrimination, although long-term performance may require disease-specific recalibration.

## 6. Focal and Segmental Glomerulosclerosis and Minimal Change Disease

### 6.1. Histological Prognostic Factors

Prospective multicenter cohorts from NEPTUNE and CureGN consistently demonstrate that chronic structural injury is the dominant histologic determinant of renal outcome in FSGS and MCD [47,48,49,50,51]. Chronic glomerular lesions—including global and segmental sclerosis—and tubulointerstitial damage were the strongest predictors of composite renal endpoints, whereas podocyte foot process effacement showed limited prognostic value. A higher proportion of morphologically abnormal glomeruli independently increased progression risk, while a greater percentage of minimally affected glomeruli predicted favorable outcomes [47].

Across independent cohorts, the quantitative burden of glomerulosclerosis—expressed as segmental sclerosis ratio or proportion of globally sclerosed glomeruli—consistently predicted kidney failure independent of FSGS subtype [48,49]. In a cohort of 206 patients stratified by segmental sclerosis ratio (≤15% vs. >15%), higher sclerosis burden was independently associated with progression to ESKD (HR 2.31, 95% CI 1.02–5.21) and correlated with more severe tubulointerstitial injury [48]. Similarly, in an 82-patient cohort, greater global sclerosis was associated with lower baseline eGFR, whereas increasing segmental sclerosis burden predicted faster annual eGFR decline (−1.5 mL/min/1.73 m^2^ per year per 10% increase) [49]. Collectively, lesion extent—rather than etiologic subtype—emerged as the principal histologic driver of long-term prognosis.

In the CureGN prospective cohort (n = 650; 476 genotyped), high-risk APOL1 status was identified in 18% of participants and was independently associated with more rapid kidney function decline, with 2.75-fold higher odds of rapid eGFR slope compared with low-risk genotypes and a faster annual eGFR loss (−1.84 mL/min/1.73 m^2^/year) [50]. Progression to kidney failure was also more frequent in high-risk individuals (32% vs. 19%). However, in a pathology-defined subgroup (n = 192), high-risk APOL1 genotype was strongly associated with collapsing lesions and more severe tubulointerstitial injury, and after adjustment for histologic severity, APOL1 genotype was no longer independently predictive. Instead, advanced chronic injury—particularly IF/TA > 50%—emerged as the dominant determinant of rapid eGFR decline (OR 9.64, 95% CI 2.38–38.50), suggesting that structural chronicity mediates much of the genotype-associated risk.

Computational pathology approaches further refined risk stratification by quantifying tubular and basement membrane features that improved prognostic discrimination beyond conventional visual scoring, reinforcing the central role of quantitative tubulointerstitial remodelling in outcome prediction [51]. In NEPTUNE/CureGN cohorts, computational pathology using deep-learning analysis of PAS-stained whole-slide images refined prognostication by quantifying tubular features in patients with FSGS and MCD [51]. Selected quantitative tubular metrics independently predicted disease progression and improved discrimination beyond conventional clinical and visually scored parameters (iAUC 0.753 to 0.811), while distinct tubular basement membrane and epithelial features were associated with proteinuria remission (iAUC 0.724). These abnormalities increased with advancing IF/TA severity, underscoring the prognostic relevance of quantitative tubular remodeling.

### 6.2. Clinical and Biochemical Prognostic Factors

Proteinuria trajectory remains the principal dynamic clinical predictor of outcome in FSGS. In trial-based and observational cohorts, early reductions in proteinuria were independently associated with improved eGFR slope and lower risk of ESKD or death, even when complete remission was not achieved [52]. Among 138 participants, early reductions in proteinuria (within 26 weeks) were independently associated with improved long-term renal outcomes [52]. Each 1-unit decrease in log-UPCR was associated with a +3.9 mL/min/1.73 m^2^ per year improvement in eGFR slope and a substantially lower risk of ESKD or death (HR 0.23), effects that persisted after adjustment for complete remission. Modest reductions (20–30%) conferred clinically meaningful preservation of kidney function, supporting incremental reductions in proteinuria as a surrogate marker of benefit.

Baseline albuminuria and kidney function further refine prognosis. In a prospective analysis of 159 adults with biopsy-confirmed FSGS from the GCKD cohort (median follow-up 6.5 years), higher baseline albuminuria independently predicted adverse renal and cardiovascular outcomes [53]. UACR ≥ 0.7 g/g was associated with increased risk of the composite kidney endpoint (HR 5.27) and major adverse cardiovascular events (HR 3.37), whereas higher baseline eGFR was protective. Albuminuria—rather than total proteinuria—emerged as an independent predictor of both renal and cardiovascular risk.

Across etiologic subtypes, FSGS classification was not independently associated with kidney failure after adjustment for clinical and histologic factors [49]. Instead, male sex, lower baseline eGFR, and higher baseline proteinuria consistently predicted progression and mortality. Baseline proteinuria was also associated with a steeper decline in eGFR across subtypes, reinforcing its central role as a clinical determinant of outcome.

Age at disease onset did not significantly influence long-term renal survival, with comparable risks of ESKD and composite renal endpoints across pediatric and adult populations [54].

Complement activation has emerged as an additional biologic modifier of risk. In CureGN, glomerular C3 deposition correlated with chronic and active lesions, and urinary soluble C5b-9 independently predicted kidney disease progression (HR 1.64, 95% CI 1.03–2.60) [55]. An independent cohort confirmed markedly higher urinary sC5b-9 and C5a levels in FSGS versus MCD, with excellent discrimination, supporting complement activation as a marker of active disease rather than irreversible scarring [56].

Collectively, cumulative structural injury and dynamic proteinuria response constitute the dominant axes of prognostic stratification in FSGS and MCD, whereas etiologic subtype and genotype provide contextual refinement rather than primary risk determination.

Table 8 summarizes key prognostic determinants in FSGS identified across contemporary cohort and biomarker studies. Chronic histologic injury—particularly global and segmental sclerosis, tubulointerstitial damage, and collapsing lesions—represents the dominant driver of renal decline, with quantitative lesion burden (e.g., SSR, IF/TA extent) and computational tubular metrics refining risk discrimination. Among clinical factors, baseline proteinuria/albuminuria, eGFR, and early proteinuria reduction independently predict outcome. APOL1 high-risk genotype confers increased progression risk, although its effect is partly mediated by the severity of underlying structural injury.

### 6.3. Nomograms and Risk Scores for Prognosis

A clinicopathologic risk score for predicting progression to ESKD in FSGS was developed in a retrospective cohort of 359 patients with biopsy-proven disease [57]. The model incorporated baseline eGFR, proteinuria, mean arterial pressure, serum IgG, and a semiquantitative tubulointerstitial lesion score, integrating clinical and structural parameters. In multivariable Cox analysis, the score demonstrated good discrimination for 5-year ESKD risk (AUC 0.86–0.91) and stratified patients into risk categories with progressively lower renal survival.

## 7. Prognostic Domains and Translational Gaps in Glomerulonephritis

GN spans five interrelated domains—histology, dynamic clinical trajectories, disease-specific biomarkers, predictive models, and implementation. Each domain contributes distinct information, yet their value emerges primarily through integration rather than isolated interpretation. Histologic scoring systems remain foundational because they quantify structural injury; however, they are limited by observer variability, sampling constraints, disease-specific applicability, and evolving classifications. Clinical endpoints such as proteinuria and eGFR are reproducible and universally available, but their prognostic meaning depends on disease context and longitudinal behavior. Biomarkers—including anti-PLA2R antibodies, urinary β2-microglobulin, complement activation products, and genetic susceptibility factors—offer mechanistic specificity, although many lack assay standardization, prospective validation, and seamless incorporation into routine care. Risk prediction models, ranging from traditional nomograms to machine-learning algorithms, frequently demonstrate strong discriminative performance in retrospective cohorts, yet translation into everyday practice remains inconsistent due to calibration challenges, interpretability concerns, and limited external validation. Table 9 synthesizes these domains and their respective strengths and translational constraints.

Importantly, these tools do not operate at a single decision level and should not be interpreted interchangeably. Rather, they function across complementary layers of risk assessment that converge in clinical and research decision-making.

At the structural level, chronicity markers—particularly interstitial fibrosis, tubular atrophy, and global glomerulosclerosis—define the ceiling of reversibility at the time of biopsy. These lesions represent accumulated nephron loss and provide essential context for therapeutic expectations, effectively calibrating treatment intensity according to the burden of irreversible damage.

Superimposed on this structural substrate are dynamic clinical trajectories, including sustained or time-averaged proteinuria, remission durability, and early eGFR slope. Unlike static histologic findings, these parameters evolve longitudinally and therefore capture the ongoing burden of injury. Across GN subtypes, reduced baseline kidney function and subsequent eGFR trajectory consistently predict progression in MN, IgAN, C3G/MPGN, and FSGS/MCD, with early eGFR slope particularly validated as a surrogate endpoint in IgAN. Similarly, sustained proteinuria burden predicts adverse outcomes across entities and serves as a practical marker of treatment response and relapse, particularly in MN and FSGS/MCD.

Layered onto structural reserve and dynamic activity are disease-specific mechanistic biomarkers. Anti-PLA2R antibodies in MN, complement activation markers in C3G/MPGN, and genetic susceptibility factors such as APOL1 variants in FSGS refine biologic stratification by identifying active pathogenic pathways. These markers inform mechanism-targeted therapy and may enable biologically enriched clinical trial enrollment. However, their prognostic impact is frequently contextual: once substantial chronic tubulointerstitial injury is established, structural damage often predominates over genetic susceptibility in determining long-term renal survival. In this sense, chronic histologic injury—particularly interstitial fibrosis, tubular atrophy, and global glomerulosclerosis—emerges as a convergent final pathway of irreversible nephron loss across GN subtypes.

Integrative risk prediction models and machine-learning approaches occupy a complementary layer. By synthesizing structural, dynamic, and molecular variables into composite probability estimates, these tools aim to formalize risk communication and stratification. Established models such as the IgAN Prediction Tool demonstrate clear clinical applicability, whereas many machine-learning algorithms remain exploratory due to incomplete external validation, uncertain calibration across populations, and lack of prospective implementation studies demonstrating improved patient outcomes. Importantly, even when validation is reported, models are frequently tested in cohorts that differ by ethnicity, baseline risk, access to therapies, and endpoint definitions, which may substantially affect calibration and transportability across real-world settings.

**Figure 1:**
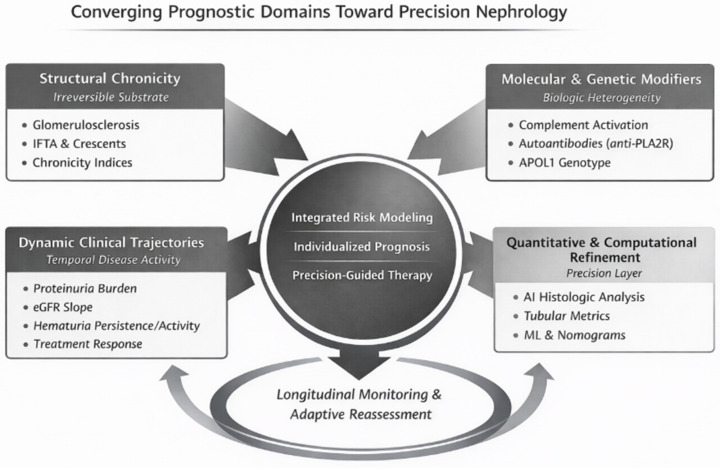
Renal outcome is determined by the interaction of structural chronicity, dynamic clinical trajectories, molecular and genetic modifiers, and quantitative computational refinement. These multidimensional inputs converge into integrated risk modeling, enabling individualized prognosis and precision-guided therapeutic decision-making. Continuous longitudinal reassessment further refines risk estimates over time.

From a translational perspective, prognostic stratification should guide individualized management rather than remain descriptive. In routine clinical practice, validated tools—including baseline eGFR, longitudinal proteinuria monitoring, eGFR slope assessment, chronicity lesion quantification, anti-PLA2R antibody titers in MN, and established IgAN risk models—can directly inform monitoring intensity, therapeutic escalation, and long-term counseling. In parallel, these same domains support clinical trial design and evaluation: dynamic proteinuria reduction and eGFR slope are increasingly used as trial outcomes and surrogate readouts of treatment effect; biomarkers can enable mechanistic enrichment and early response monitoring (e.g., anti-PLA2R dynamics in MN or complement biomarkers in C3G/MPGN); and risk models can support enrichment strategies by identifying high-risk patients more likely to accrue endpoints. In contrast, many omics-derived biomarker panels and advanced computational approaches currently serve primarily research functions, supporting hypothesis generation and exploratory stratification rather than routine bedside decision-making. The integrative relationship between structural reserve, evolving clinical trajectories, biological drivers, and probabilistic modeling is schematically illustrated in Figure 1, emphasizing how these domains converge to inform both individualized care and interventional trial design.

Despite these advances, the current evidence base remains subject to important limitations. Most studies are retrospective and derived from biopsy-based cohorts, introducing selection bias and limiting generalizability to unbiopsied or milder cases. Substantial heterogeneity exists in follow-up duration, baseline disease severity, and endpoint definitions, with renal outcomes variably defined as ESKD, doubling of serum creatinine, or ≥40–50% eGFR decline, and response thresholds inconsistently applied. Treatment exposure acts as a major confounder and effect modifier, particularly across evolving therapeutic eras characterized by optimized RAAS blockade, increasing use of rituximab in MN, and the emergence of targeted agents such as SGLT2 inhibitors, endothelin receptor antagonists, and complement inhibitors. Furthermore, many prognostic cohorts and validation studies were developed in relatively homogeneous populations or selected healthcare settings, and differences in ethnicity, disease phenotype, supportive care, and treatment patterns may limit transferability and calibration across diverse geographic regions.

Methodological constraints also affect predictive models and biomarker studies. Several scores originate from single-center cohorts, rely on non-uniform endpoints, and incorporate variables not universally available in routine care. External validation and calibration are inconsistently reported, and validation cohorts may differ substantially from derivation cohorts in baseline risk and treatment exposure. Machine-learning approaches introduce additional concerns, including overfitting, limited transparency in feature selection, restricted interpretability despite explainability methods (e.g., SHAP), and absence of prospective studies demonstrating clinical utility. Biomarker investigations are often limited by small sample sizes, heterogeneous assay platforms, and uncertain incremental value beyond established clinical and histologic parameters. Addressing these challenges will require prospective multicenter validation, standardized endpoint definitions, harmonized laboratory methodologies, and pragmatic decision-support tools capable of integrating structural injury, dynamic trajectories, and biologic drivers into clinically actionable strategies.

Viewed through this framework, integration in precision nephrology is not merely the sequential presentation of domains but the coordinated interaction between structural reserve, evolving injury burden, disease-specific biology, and probabilistic modeling—each contributing at a distinct yet interdependent decision layer.

## 8. Conclusions

Prognostic stratification in primary glomerulonephritis has progressed from isolated clinical markers to an integrated framework combining structural chronicity, dynamic clinical trajectories, disease-specific biomarkers, and quantitative risk modeling. Across GN subtypes, chronic histologic injury defines the ceiling of reversibility, while sustained proteinuria burden and eGFR slope capture ongoing disease activity and treatment response. Biomarkers refine mechanistic stratification, and validated prediction tools formalize individualized risk estimation and support trial enrichment strategies.

The key challenge is no longer identifying additional markers but integrating these domains coherently across clinical and research decision layers. Future advances require prospective validation, harmonized endpoints, standardized biomarker platforms, and implementable decision-support systems capable of translating predictive accuracy into meaningful improvements in patient outcomes.

## Figures and Tables

**Table 1:** Summary of key histological and biomarker-based prognostic factors in membranous nephropathy.

Prognostic Factors	Endpoint(s)	Key Prognostic Findings
**FSGS**	eGFR, remission	Lower eGFR; reduced remission; component of FSTIV score
	≥50% eGFR decline/ESKD	Typical lesions are independently associated with progression (HR 2.47); atypical lesions are not prognostic
**TID**	Remission	TID+ associated with worse baseline renal parameters and lower CR (16.7% vs. 35.0%)
	Renal endpoints	Independently associated with renal events (HR 2.12)
	ESKD; >50% eGFR decline	Severe injury (>50%) associated with progression (HR 25.77)
**Vascular lesions (arteriolosclerosis)**	Composite renal events	Associated with reduced renal survival, higher proteinuria, IF
**GBM thickness/EC stage**	Complete remission	Increased GBM thickness independently reduced the likelihood of CR (HR per SD 0.58)
	Single-nephron GFR	Advanced EC stages are independently associated with reduced SNGFR
**Anti-PLA2R positivity**	Remission	Lower CR (OR 0.50) and spontaneous remission (OR 0.30)
**Anti-PLA2R titer**	Remission	High titers associated with reduced remission
**Microscopic hematuria**	Relapse; renal progression	Baseline, persistent and worsening hematuria—Associated with relapse and ≥40% eGFR decline/ESKD
**β2-microglobulin (urinary)**	Renal progression	AUC 0.80
**α1-microglobulin (urinary)**	Renal progression	Comparable performance to β2-microglobulin (AUC 0.79)
**AKI during follow-up**	ESKD, renal survival	Associated with reduced renal survival
**CXCL13 (urinary)**	Remission	Lower baseline levels predicted response
**GDF15 (plasma/urine)**	Treatment response	Decreasing levels associated with remission
**TWEAK, TNF-α**	Disease activity	Correlated with proteinuria, eGFR, anti-PLA2R

**Table 2:** Prognostic models and risk scores in membranous nephropathy.

Model	Variables	Performance	Clinical Role
**Toronto Risk Score**	Proteinuria, sCr (6–12 months)	AUC~0.78	Longitudinal risk stratification
**Shanghai risk score**	Age, eGFR, proteinuria	AUC 0.83	Baseline progression risk
**FAR-based model**	Fibrinogen/albumin ± anti-PLA2R	AUC up to 0.77	non-remission prediction
**LightGBM ML model**	Clinical + bio + histology	AUC 0.892	Individualized risk
**PLA2R-based nomograms**	PLA2R, proteinuria, albumin	AUC 0.80–0.87	Clinically applicable tools

**Table 3:** Histological and clinical prognostic factors in C3 glomerulopathy.

Prognostic Factors	Endpoint(s)	Key Prognostic Findings
**Interstitial fibrosis/tubular atrophy**	Composite renal outcome (>30% eGFR decline, doubling of serum creatinine, ESKD, or RRT)	Independently associated with progression
**Segmental sclerosis**		eGFR decline, doubling of serum creatinine, and ESKD
**Cellular/fibrocellular crescents**		Associated with increased risk
**Endocapillary hypercellularity**	Proteinuria	correlated with proteinuria severity
**GBM double contours**		
**C3G Histologic Index—chronicity score (TCS)**	Renal survival	TCS ≥ 4 associated with lower 3-year renal survival (72% vs. 91%), but not independent in multivariate analysis
**Chronic histologic lesions (IF, glomerulosclerosis)**	ESKD, advanced CKD	Chronic lesions were the strongest predictors of renal progression
**Activity and chronicity index scores**	Renal deterioration	Independently associated with outcome
**Baseline eGFR**	ESKD, CKD progression	Lower baseline eGFR consistently predicted poor renal outcome
**Hemoglobin**	Renal survival	Lower levels independently associated with adverse outcome (HR 0.67)
**Doubling of proteinuria**	Renal failure	≈2.5-fold increased risk
**≥50% reduction in proteinuria**	Renal deterioration	Associated with lower risk, including early reduction
**>30% reduction at 6 months**	Renal survival	Predicts improved outcomes
**Proteinuria < 1 g/day (long-term)**	Renal survival	Associated with improved long-term prognosis

**Table 4:** Prognostic relevance of histopathologic classification in C3G and MPGN.

Histological Patterns	Key Findings	Prognostic Relevance
**C3GN vs. DDD**	No significant differences in renal outcomes despite differing activity/chronicity scores	Overall histologic burden more informative than subtype
**Immune-complex associated MPGN vs. C3G**	Comparable clinical presentation, histology, and renal outcomes	Limited prognostic discrimination by etiologic category
**Classical MPGN pattern**	Present in only ~34% of cases	Limited independent prognostic value

**Table 5:** Complement-related biomarkers and their prognostic significance in C3 glomerulopathy.

Biomarker	Prognostic Implication
**Low serum C3**	Associated with increased risk of progression to ESKD
**Elevated soluble C5b-9 (sC5b-9)**	Associated with adverse renal outcomes
**High C3 nephritic factor (C3NeF) activity**	Associated with adverse renal outcomes

**Table 6:** Histologic and clinical prognostic factors in IgA nephropathy.

Prognostic Factors (Oxford/Extended)	Endpoint(s)	Key Prognostic Findings
**Mesangial hypercellularity (M1)**	Kidney failure	Associated with progression vs. M0 (HR ≈ 1.7)
**Segmental glomerulosclerosis (S1)**		Associated with progression (HR 1.8; 95% CI 1.4–2.4)
**Tubular atrophy/interstitial fibrosis (T1/T2)**		HR 3.2; 95% CI 1.8–5.6
**Endocapillary hypercellularity (E1)**		Not independently predictive; high heterogeneity
**Crescents (C1/C2)**	Kidney failure	Associated with progression (HR ≈ 1.9)
**Crescent burden (>10%, >25%)**	ESKD, composite outcome	Dose–response relationship; **C2 (≥25%) highest risk**, independent of treatment
**Oxford MEST-C score (integrated)**	10-yr renal survival	Higher grades associated with reduced survival
**Global injury patterns (sclerosing, crescentic)**	eGFR decline, ESKD	Sclerosing (HR 2.1) and crescentic (HR 3.6) patterns predicted poor outcome
**Mesangial C3 deposition**	Renal survival	Independently associated with adverse outcome (HR ≈ 3.3)
**Time-averaged proteinuria (TA-P)**	Kidney failure, death	Dose–response association
**Each 10% TA-P reduction**		11% risk reduction
**Time-weighted average proteinuria (TWAP)**	eGFR slope	Higher TWAP independently accelerated eGFR loss
**Proteinuria < 0.3 g/g**	Long-term progression	Associated with slower eGFR decline
**1-year eGFR slope**	Composite renal outcome	Independent surrogate marker (R^2^ ≈ 0.86)
**Initial microscopic hematuria**	ESKD	Associated with increased risk (RR 1.87)
**Macroscopic hematuria**		Inversely associated with risk (RR 0.68)
**Persistent hematuria**	ESKD/≥50% eGFR decline	Associated with progression

**Table 7:** Prognostic risk models applied to IgA nephropathy.

Model	Variables	Performance	Clinical Role
**International IgAN Prediction Tool**	Clinical + MEST ± race	C-statistic~0.81–0.82	disease-specific risk prediction
**External validations (China, elderly)**	Same	C ≈ 0.79–0.82; variable calibration	Validation across populations
**KFRE (generic CKD)**	Age, sex, eGFR, UACR	AUC~0.78 (5 yrs)	Acceptable short-term, weaker long-term
**Updated KFRE (IgAN-adapted)**	Same variables	AUC up to 0.84	Adapted prediction in IgAN

**Table 8:** Histologic and clinical prognostic markers in focal segmental glomerulosclerosis.

Histologic Feature	Endpoint(s)	Prognostic Significance
**Extent of chronic glomerular lesions** (global sclerosis, segmental sclerosis, collapse, periglomerular fibrosis)	≥40% eGFR decline or kidney failure	Independently associated with progression; higher lesion burden higher risk
**Tubulointerstitial injury (IF/TA, inflammation, ATI)**	Renal progression; proteinuria remission	Dominant predictor of progression; inversely associated with remission
**Percentage of glomeruli with minimal/no lesions**	Renal progression	Independent favorable prognostic marker, even beyond MCD diagnosis
**Segmental sclerosis ratio (SSR > 15%)**	ESKD	Independent predictor of ESKD (HR 2.31; 95% CI 1.02–5.21)
**Global glomerulosclerosis burden**	Kidney failure; baseline eGFR	–4.8 mL/min/1.73 m^2^ eGFR per 10% increase
**Segmental sclerosis burden**	Annual eGFR slope	–1.5 mL/min/1.73 m^2^/year per 10% increase
**Collapsing FSGS lesions**	eGFR slope	Associated with rapid progression; enriched in APOL1 high-risk
**Severe IF/TA (>50%)**	Rapid eGFR decline	Strong independent predictor (OR 9.64; 95% CI 2.38–38.50)
**Computational tubular features** (TBM thickness, epithelial area, nuclear–lumen distance)	Renal progression; remission	Improved prognostic discrimination beyond visual scoring (iAUC to 0.81)
**Early proteinuria reduction**	eGFR slope; ESKD/death	1-log UPCR reduction +3.9 mL/min/year; HR 0.23
**Modest proteinuria reduction (20–30%)**	Renal survival	Associated with improved outcomes
**Baseline albuminuria (UACR ≥ 0.7 g/g)**	Kidney failure; MACE	HR 5.27 for renal endpoint
**Baseline eGFR**	Renal + cardiovascular outcomes	Independently protective
**Baseline proteinuria**	Kidney failure; eGFR slope	Independently predictor across subtypes
**Etiologic FSGS subtype**	Kidney failure	Not independently predictive after adjustment
**Age at onset (child vs. adult)**	ESKD; composite renal endpoint	Similar long-term renal outcomes across age groups
**APOL1 high-risk genotype**	eGFR slope; kidney failure	OR 2.75 for rapid progression; effect attenuated after histologic adjustment
**APOL1 genotype (pathology-adjusted)**	eGFR slope	Effect attenuated after adjustment for IF/TA severity
**Glomerular C3 deposition**	Renal progression	Associated with structural injury
**Urinary sC5b-9/C5a**	≥40% eGFR decline or ESKD	Independent predictor (HR 1.64); discriminates FSGS vs. MCD

**Table 9:** Summary of key prognostic domains in glomerulonephritis, including histologic markers, emerging biomarkers, clinical endpoints, predictive models, and barriers to clinical implementation.

Domain	Knowns	Unknowns	Future Directions
**Histopathology**	• Chronic lesions (IF/TA, sclerosis) predict long-term renal decline across GN subtypes. • In IgAN, M, S, T, and crescent burden consistently predict outcomes. • In MPGN/C3G, chronicity and crescents drive progression.	• How modifiable chronic lesions truly are. • Standardization and clinical integration of computational pathology. • Optimal thresholds for lesion severity across diseases.	• Prospective validation of digital/quantitative pathology. • Unified chronicity scoring across GN. • Linking histologic signatures with treatment response.
**Biomarkers**	• Anti-PLA2R level predicts remission in MN. • Persistent hematuria predicts relapse/progression in MN. • LMW urinary proteins signal tubular injury and future decline.	• Lack of standardized cutoffs. • Limited data on biomarker dynamics over time. • Unclear incremental value over histology + proteinuria.	• Development of multi-marker panels. • Validation of complement biomarkers in larger cohorts. • Biomarker-guided treatment algorithms.
**Clinical Trajectories**	• Time-averaged proteinuria is a major predictor in all GN subtypes. • Even modest proteinuria reductions improve prognosis. • AKI episodes worsen outcomes in MN and likely in others. • Baseline eGFR and albuminuria remain strong predictors.	• Best definitions for proteinuria-response trajectories. • Prognostic impact of transient vs. persistent AKI. • Role of early eGFR slope as a surrogate endpoint.	• Disease-specific proteinuria targets. • Trials focused on AKI prevention. • Predictive models integrating real-time clinical data.
**Risk Models & ML**	• IgAN International Prediction Tool shows strong performance. • Composite clinical–pathology scores predict ESKD in FSGS.	• Limited external validation. • Real-world usability and interpretability. • Sparse incorporation of biomarker dynamics.	• Explainable ML models using multimodal data. • External validation in diverse populations. • Clinically deployable digital tools.
**Therapeutic Response Prediction**	• Anti-PLA2R trajectories reflect treatment response in MN. • Early proteinuria reduction predicts benefit in FSGS.	• Mechanisms of treatment resistance. • Predictors of response to emerging therapies (SGLT2i, complement inhibitors). • Lack of validated biomarker thresholds for escalation.	• Prospective biomarker-stratified trials. • Pathway-based predictors of steroid/IST resistance. • Integration of pharmacogenomics with biomarker-based risk tools.

## Data Availability

Not applicable.

## Abbreviations

The following abbreviations are used in this manuscript:

Abbreviation	Full Term
aFSL	atypical Focal Segmental Lesion(s)
AIC	Akaike Information Criterion
AKI	Acute Kidney Injury
APOL1	Apolipoprotein L1
AST	Aspartate Aminotransferase
AUC	Area Under the Curve
BUN	Blood Urea Nitrogen
C3G	C3 Glomerulopathy
C3G-HI	C3 Glomerulopathy Histologic Index
C3GN	C3 Glomerulonephritis
C3NeF	C3 Nephritic Factor
CD-H	Cumulative Duration of Hematuria
CI	Confidence Interval
CKD	Chronic Kidney Disease
CR	Complete Remission
CXCL13	C-X-C Motif Chemokine Ligand 13
DDD	Dense Deposit Disease
EC stage	Ehrenreich–Churg Stage
eGFR	Estimated Glomerular Filtration Rate
EM	Electron Microscopy
ESKD	End-Stage Kidney Disease
FAR	Fibrinogen-to-Albumin Ratio
FSGS	Focal Segmental Glomerulosclerosis
FSTIV	Composite score (FSGS, TA, IF, VH)
GBM	Glomerular Basement Membrane
GDF15	Growth Differentiation Factor 15
GFR	Glomerular Filtration Rate
GN	Glomerulonephritis
HR	Hazard Ratio
IDI	Integrated Discrimination Improvement
IF	Interstitial Fibrosis
IF/TA	Interstitial Fibrosis and Tubular Atrophy
IgAN	IgA Nephropathy
iAUC	Integrated Area Under the Curve
KDIGO	Kidney Disease: Improving Global Outcomes
KFRE	Kidney Failure Risk Equation
LightGBM	Light Gradient Boosting Machine
MACE	Major Adverse Cardiovascular Event
MCD	Minimal Change Disease
MEST-C	Mesangial, Endocapillary, Segmental, Tubular, Crescents (Oxford Score)
ML	Machine Learning
MN	Membranous Nephropathy
MPGN	Membranoproliferative Glomerulonephritis
naFSL	Not atypical Focal Segmental Lesion(s)
NRI	Net Reclassification Improvement
NSAIDs	Non-Steroidal Anti-Inflammatory Drugs
OR	Odds Ratio
PAS	Periodic Acid–Schiff
PLA2R	Phospholipase A2 Receptor
RAAS	Renin–Angiotensin–Aldosterone System
RaDaR	Registry of Rare Kidney Diseases
RBC/HPF	Red Blood Cells per High Power Field
RRT	Renal Replacement Therapy
sC5b-9	Soluble Membrane Attack Complex
sCr	Serum Creatinine
sHR	Subdistribution Hazard Ratio
SNGFR	Single-Nephron Glomerular Filtration Rate
SSR	Segmental Sclerosis Ratio
STARMEN	Steroids and Tacrolimus vs. Rituximab in Membranous Nephropathy Trial
TA	Tubular Atrophy
TA-H	Time-Averaged Hematuria
TA-P	Time-Averaged Proteinuria
TBM	Tubular Basement Membrane
TCS	Total Chronicity Score
TID	Tubulointerstitial Damage/Lesions
TNF-α	Tumor Necrosis Factor-alpha
TWEAK	TNF-like Weak Inducer of Apoptosis
TWAP	Time-Weighted Average Proteinuria
UACR	Urinary Albumin-to-Creatinine Ratio
UPCR	Urinary Protein-to-Creatinine Ratio
VH	Vascular Hyalinosis
